# Trends, disparities, and forecasts of peripheral artery disease- and sepsis-associated mortality in the United States, 1999–2023

**DOI:** 10.3389/fmed.2026.1844478

**Published:** 2026-07-02

**Authors:** Zhan Chen, Ruimin Lin, Yue Zhang

**Affiliations:** 1 Department of Vascular Surgery, Beijing Haidian Hospital, Beijing, China; 2 Department of Scientific Research, Beijing Haidian Hospital, Beijing, China

**Keywords:** CDC WONDER, forecasting, health disparities, mortality, peripheral artery disease, rural–urban disparity, sepsis

## Abstract

**Background:**

Peripheral artery disease (PAD) and sepsis may co-occur as a high-risk mortality phenotype, yet U.S. surveillance has largely assessed them separately. We examined long-term trends, disparities, geographic heterogeneity, and near-term forecasts for mortality with concurrent documentation of PAD and sepsis in the United States.

**Methods:**

We conducted a retrospective, population-based mortality study using CDC WONDER Multiple Cause-of-Death data (1999–2023) among adults aged ≥45 years. PAD–sepsis–associated deaths were defined by co-listing of PAD (ICD-10 I70.2, I73.9) and sepsis (A40.0–A40.3, A40.8–A40.9, A41.0–A41.5, A41.8–A41.9) anywhere on the death certificate. We estimated annual age-adjusted mortality rates (AAMRs) per 100,000, assessed trends with joinpoint regression, and evaluated disparities by sex, age, race/ethnicity, Census region, and rural–large metropolitan status (rate ratio [RR], rate difference [RD]). Forecasts for 2024–2033 used ARIMA, ARIMAX with a prespecified COVID-19 pulse (2020–2021), and a 2010–2023 joinpoint-aware ARIMA approach.

**Results:**

From 1999–2023, 101,369 deaths met criteria; the period-average AAMR was 3.28 (95% CI, 3.26–3.30). AAMR declined from 4.46 (1999) to 3.06 (2023), with a nadir in 2019 (2.91), a transient increase in 2020–2021 (3.15–3.22), and attenuation in 2022–2023. Mortality was higher in males than females (period-average AAMR 3.96 vs. 2.76). Rates increased with age; crude mortality declined in adults aged ≥85 years (34.59 in 1999 to 16.90 in 2023; AAPC −3.20%/year) but increased in ages 45–54 (0.35 to 0.44; AAPC +0.91%/year). Rural–large metropolitan disparities emerged after 2010 (2010–2019 mean RR 1.21) and peaked in 2018 (RR 1.37). NH Black individuals had the highest burden (period-average AAMR 6.48 vs. 3.02 in NH White) but declined from 11.16 (1999) to 5.74 (2023; AAPC −2.84%/year). Forecasts for 2024–2033 suggested no rapid escalation (ARIMA: 3.05 to 2.95; ARIMAX: 3.06 to 3.09; 2010–2023 joinpoint-aware approach: 3.12 to 3.23).

**Conclusions:**

PAD-sepsis-associated mortality declined overall with a transient increase during 2020–2021, while inequities persisted, including sustained rural excess after 2010 and an unfavorable trajectory in adults aged 45–54 years. Projections through 2033 indicated relative stability, supporting continued surveillance and integrated prevention and care strategies.

## Background

Peripheral artery disease (PAD), a common manifestation of systemic atherosclerosis, is associated with substantial morbidity, limb loss, and premature mortality (1, 2). Although frequently framed as a limb-limited condition, PAD reflects diffuse vascular disease and clustered cardiometabolic comorbidity—including diabetes and chronic kidney disease—that may reduce physiologic reserve and increase susceptibility to acute illness (1). Sepsis remains a leading cause of death in hospitals and a major driver of health care utilization in the United States, with outcomes shaped by host vulnerability, timely recognition and treatment, and the capacity of acute-care systems (3–6). The overlap between PAD and sepsis is therefore clinically plausible: impaired tissue perfusion, microvascular dysfunction, chronic inflammation, and recurrent skin or foot breakdown may increase the risk of infection and progression to severe systemic illness, while sepsis-related hemodynamic stress can precipitate cardiovascular decompensation and exacerbate ischemic complications in patients with established atherosclerotic disease (1, 6, 7). From an infectious disease perspective, PAD may increase susceptibility to severe infection through several interconnected pathways. Chronic arterial insufficiency may reduce tissue oxygenation, delay wound healing, compromise skin and soft-tissue barrier integrity, and limit both local immune responses and antimicrobial delivery to infected tissue (1, 7). In patients with diabetes, neuropathy and repetitive trauma may further promote ulcer formation, while ischemia can impair local host defense and facilitate persistent or recurrent infection. Once established, limb and soft-tissue infections in poorly perfused tissue may become polymicrobial, difficult to eradicate, and more likely to progress to bacteremia, systemic inflammation, and sepsis. In this context, antimicrobial resistance and empirical antibiotic challenges in diabetic foot infections may further increase the risk of treatment failure and progression to limb-threatening or systemic complications (8). Therefore, mortality with co-documented PAD and sepsis is directly relevant to infectious disease practice because it reflects the intersection of host vascular vulnerability, pathogen burden, antimicrobial treatment challenges, early infection recognition, and timely source control (6).

Despite this biologic and clinical coherence, population surveillance has typically examined PAD and sepsis in isolation. Prior studies have characterized trends in PAD-related outcomes (including amputation, hospitalization, and PAD-attributed mortality) or have evaluated sepsis mortality patterns and disparities separately (1, 9). Mortality in which both PAD and sepsis are documented may represent a distinct, high-risk phenotype that is not captured by single-condition endpoints and may be particularly sensitive to changes in chronic disease prevention, acute-care delivery, and health system strain. Moreover, both PAD and sepsis show marked disparities by age, sex, race and ethnicity, and geography, raising the possibility that favorable national trends may conceal persistent or widening inequities (1, 9, 10). Such concerns are especially salient for rural populations, where higher cardiometabolic burden and constrained access to specialty vascular services and critical care may compound risk, and for racial and ethnic groups facing structural barriers to preventive and timely acute care (9, 10).

Clarifying long-term patterns at the PAD–sepsis interface has direct implications for clinical practice, health system planning, and equity-focused public health strategies. If mortality involving both conditions is declining overall yet worsening in specific subgroups or clustering geographically, these signals could inform targeted approaches that integrate vascular risk reduction with infection prevention, early recognition, and timely treatment, including efforts to reduce delays in evaluation, antibiotic administration, and definitive vascular care (1, 6).

We therefore conducted a retrospective, population-based analysis of U.S. death certificate data from 1999 through 2023 using the CDC WONDER Multiple Cause-of-Death database (11). We quantified national and state-level trends in mortality with concurrent documentation of PAD and sepsis, identified temporal inflection points, and assessed disparities by sex, age group, race and ethnicity, region, and rural–metropolitan status. We also generated near-term forecasts for 2024–2033 using complementary time-series models, including a prespecified COVID-19 intervention specification, to contextualize recent perturbations and inform expectations for this clinically important intersection of chronic atherosclerotic disease and severe infection.

## Methods

### Data source and study design

We conducted a retrospective, population-based mortality study using the Centers for Disease Control and Prevention (CDC) WONDER Multiple Cause-of-Death (MCOD) database from January 1, 1999, through December 31, 2023. The MCOD files are based on U.S. death certificates and include one underlying cause of death and up to 20 additional multiple (contributing) causes coded using the International Classification of Diseases, Tenth Revision (ICD-10), with population denominators derived from U.S. Census-based estimates to support annual rate calculations (12).

### Study population and case definition

Analyses were restricted to decedents aged ≥45 years and summarized using 10-year age groups (45–54, 55–64, 65–74, 75–84, and ≥85 years). PAD- and sepsis-associated mortality was defined as deaths in which both PAD and sepsis were listed anywhere on the death certificate (as underlying or contributing causes), consistent with the “any-mention” framework supported in MCOD queries. PAD was identified using ICD-10 codes I70.2 and I73.9, and sepsis using A40.0–A40.3, A40.8–A40.9, A41.0–A41.5, and A41.8–A41.9. I70.2 and I73.9 were selected a priori to capture lower-extremity peripheral arterial/peripheral vascular disease as documented on death certificates. Broader atherosclerosis codes, including I70.0, I70.1, I70.8, and I70.9, were not included because they primarily denote aortic, renal, other, or generalized/unspecified atherosclerosis rather than peripheral limb arterial disease. I73.1 for thromboangiitis obliterans/Buerger's disease was also not included because it represents a distinct non-atherosclerotic inflammatory vasculopathy. This conservative definition was intended to improve PAD/PVD phenotype specificity while minimizing heterogeneity from non-peripheral atherosclerosis and non-atherosclerotic vasculopathies. CDC WONDER suppresses strata representing 0–9 deaths; suppressed cells were treated as missing and excluded from analyses requiring those strata (13).

We selected age ≥45 years a priori because PAD-related mortality is uncommon at younger ages and small counts increase CDC WONDER suppression and instability in stratified estimates.

### Variables and outcomes

Race/ethnicity categories followed U.S. Office of Management and Budget (OMB) standards (14) and were operationalized as non-Hispanic (NH) White, NH Black or African American, Hispanic/Latino, and NH Other (American Indian/Alaska Native and Asian/Pacific Islander groups). Geographic variables included U.S. Census region, state, and urbanization based on the 2013 National Center for Health Statistics (NCHS) Urban–Rural Classification Scheme for Counties as implemented in CDC WONDER (15).

The primary outcome was the annual age-adjusted mortality rate (AAMR) per 100,000 population for PAD- and sepsis-associated mortality. Secondary outcomes included age-specific crude mortality rates (CMR) per 100,000 population and measures of rural–urban disparity. For the primary disparity analysis, we compared rural (nonmetropolitan) areas with large metropolitan areas and summarized annual disparities using the rate ratio (RR; rural/large metropolitan) and rate difference (RD; rural–large metropolitan, per 100,000 population). ICD-10 code lists and definitions of analytic strata (including race/ethnicity, region, and urbanization) are provided in Supplementary Table S1.

### Statistical analysis

Annual AAMRs and corresponding 95% confidence intervals (CIs) were estimated using direct standardization to the 2000 U.S. standard population, consistent with commonly used federal/NCHS guidance for age-adjusted death rates (16). Age adjustment was applied for overall and non-age subgroup comparisons to account for differences in age structure across populations and over time. Age-stratified analyses were reported as crude mortality rates rather than age-adjusted rates because each stratum represented a predefined 10-year age group. These crude rates reflect the directly observed age-specific mortality burden within each age category, whereas further age adjustment within fixed age strata was not necessary and could reduce interpretability of the age gradient.

Temporal trends in annual AAMRs (1999–2023) were evaluated using joinpoint regression. Joinpoint models were fit to log-linear AAMR trends using the National Cancer Institute Joinpoint Regression Program (17). The statistical significance of changepoints was assessed using Monte Carlo permutation tests (two-sided α = 0.05; 4,499 permutations), allowing up to four joinpoints. The COVID-19 period was prespecified as 2020–2021 and highlighted in figures to contextualize pandemic-era disruptions. The maximum of four joinpoints was chosen to balance flexibility with overfitting risk given a 25-year annual time series. We report segment-specific annual percent change (APC) for each joinpoint segment and the average annual percent change (AAPC) over the full study period.

Rural–metropolitan disparity was assessed annually using RR and RD with 95% CIs. To characterize geographic heterogeneity, we visualized state-by-year AAMRs as a heat map and ordered states using hierarchical clustering with Ward linkage and Euclidean distance (18).

To examine near-term future patterns, we forecasted annual AAMRs for 2024–2033 using three complementary time-series specifications. Stationarity was assessed using the augmented Dickey–Fuller framework (19), and differencing was applied as needed. First, we fit a baseline autoregressive integrated moving average (ARIMA) model following Box–Jenkins methodology (20); candidate ARIMA(p,d,q) structures were compared using the corrected Akaike information criterion (AICc) (21). Model adequacy was evaluated using residual autocorrelation diagnostics (ACF/PACF) and the Ljung–Box test (22). Second, we fit an ARIMAX intervention model incorporating a prespecified pulse indicator for the COVID-19 period (coded 1 for 2020–2021 and 0 otherwise); for forecasting, the pulse term was set to 0 for all years 2024–2033. Third, we implemented a joinpoint-aware segmented approach by fitting an ARIMA model to the 2010–2023 period and forecasting forward using a 2010–2023 ARIMA specification (ARIMA [1,0,1]). Candidate ARIMA structures were compared using information criteria, and model fit was summarized using AIC and BIC. Residual autocorrelation was evaluated using residual ACFs and Ljung–Box tests with a prespecified lag of 10.

### Software and ethics

All analyses were conducted using R (R Foundation for Statistical Computing, Vienna, Austria). Joinpoint analyses were performed using the National Cancer Institute Joinpoint Regression Program. This study used publicly available, deidentified CDC WONDER data. Institutional review board approval and informed consent were not required, and analyses complied with CDC data-use policies.

## Results

### Study population and overall burden

From 1999 through 2023, there were 101,369 deaths among adults aged 45 years or older in which both PAD and sepsis were listed anywhere on the death certificate. The inverse-variance-weighted mean AAMR over the study period (period-average AAMR) was 3.28 per 100,000 population (95% CI, 3.26–3.30; Table 1).

**Table 1:** Distribution and temporal trends in PAD- and sepsis-associated mortality among adults aged ≥45 years in the United States, 1999–2023.

Characteristic	Deaths (*n*)	AAMR per 100,000 (95% CI)	AAPC (95% CI)
Overall	101,369	3.28 (3.26, 3.30)	−1.52 (−1.66, −1.33)
Sex			
Male	50,786	3.96 (3.92, 3.99)	−1.38 (−1.65, −1.11)
Female	50,583	2.76 (2.74, 2.78)	−1.99 (−2.26, −1.72)
Age group (years)			
45–54	3,516	0.33 (0.31, 0.34)	0.91 (0.01, 2.05)
55–64	12,084	1.33 (1.30, 1.35)	0.07 (−0.27, 0.40)
65–74	23,781	3.84 (3.79, 3.88)	−0.69 (−0.96, −0.35)
75–84	31,770	8.86 (8.76, 8.96)	−1.73 (−1.92, −1.50)
85+	30,218	20.71 (20.47, 20.95)	−3.20 (−3.57, −2.86)
Race/ethnicity			
NH white	73,469	3.02 (2.99, 3.04)	−1.24 (−1.42, −1.04)
NH black	18,369	6.48 (6.39, 6.58)	−2.84 (−3.07, −2.56)
Hispanic/Latino	6,853	2.97 (2.90, 3.04)	−1.89 (−2.50, −1.13)
NH other (AI/AN, Asian/PI)	2,382	1.68 (1.61, 1.75)	−2.87 (−3.67, −1.95)
Geographic region			
Northeast	18,970	3.08 (3.04, 3.13)	−2.21 (−2.66, −1.78)
Midwest	21,946	3.17 (3.12, 3.21)	−1.40 (−1.73, −1.08)
South	41,047	3.63 (3.59, 3.66)	−1.49 (−1.76, −1.21)
West	19,406	2.96 (2.91, 3.00)	−1.46 (−1.71, −1.16)
Urban–rural status			
Large metropolitan	43,397	3.19 (3.16, 3.22)	−2.50 (−2.71, −2.22)
Medium/small metropolitan	27,286	3.34 (3.30, 3.38)	−1.42 (−1.97, −0.92)
Rural	16,578	3.58 (3.52, 3.63)	−0.80 (−1.23, −0.36)

AAMR, age-adjusted mortality rate; AAPC, average annual percent change; CI, confidence interval; PAD, peripheral artery disease; NH, non-Hispanic; AI/AN, American Indian/Alaska Native; PI, Pacific Islander.

AAMR summary values represent inverse-variance-weighted means of annual AAMRs (1999–2023) from CDC WONDER (weights = 1/SE^2^), with 95% CIs derived from the pooled variance; AAPCs (95% CIs) were obtained from joinpoint regression of annual AAMRs, and subgroup totals may not sum to the overall total because of suppressed or missing strata in CDC WONDER.

### Overall trends and sex differences

**Figure 1:**
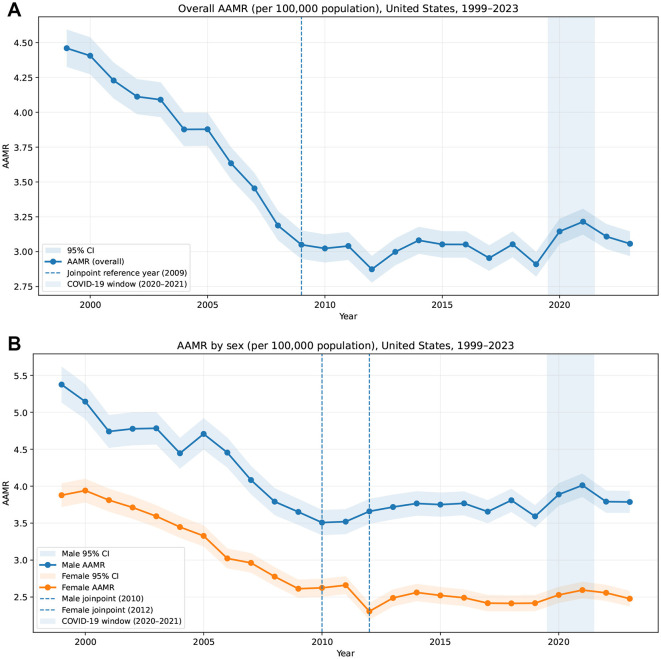
Age-adjusted mortality rate (AAMR) for PAD- and sepsis–associated mortality overall and by sex, United States, 1999–2023. **(A)** Annual overall AAMRs (per 100,000 population) are shown with 95% confidence intervals. The dashed vertical line indicates the joinpoint reference year (2009), and the shaded band marks the COVID-19 period (2020–2021). **(B)** Annual AAMRs (per 100,000 population) are shown for males and females with 95% confidence intervals. Dashed vertical lines indicate sex-specific joinpoint reference years (2010 for males and 2012 for females), and the shaded band marks the COVID-19 period (2020–2021).

The overall AAMR decreased from 4.46 per 100,000 in 1999 to 3.06 per 100,000 in 2023 (Figure 1A); the dashed line denotes the joinpoint reference year (2009). Rates reached a pre-pandemic nadir in 2019 (2.91 per 100,000) and rose during the prespecified COVID-19 period, increasing to 3.15 in 2020 and 3.22 in 2021, followed by attenuation in 2022–2023 (3.11 and 3.06, respectively; Figure 1A). Segment-specific APCs from joinpoint regression are shown in Table 2.

**Table 2:** Joinpoint segmented annual percent change (APC) in PAD- and sepsis-associated mortality rates, United States, 1999–2023.

Characteristic	Segment (years)	APC, % (95% CI)	*P* value
Overall	1999–2005	−2.39 (−3.17, −0.81)	0.020
	2005–2009	−6.16 (−8.35, −4.43)	0.008
	2009–2023	0.24 (−0.04, 0.58)	0.094
Sex			
Male	1999–2010	−3.54 (−4.63, −2.78)	< 0.001
	2010–2023	0.48 (−0.08, 1.24)	0.084
Female	1999–2012	−3.89 (−4.72, −3.31)	< 0.001
	2012–2023	0.31 (−0.48, 1.50)	0.382
Age group			
45–54	1999–2012	−1.72 (−4.78, 3.91)	0.096
	2012–2020	6.85 (−7.45, 17.59)	0.131
	2020–2023	−2.85 (−12.91, 4.99)	0.470
55–64	1999–2010	−3.07 (−4.15, −2.17)	0.018
	2010–2015	5.44 (−1.46, 10.38)	0.061
	2015–2018	−1.10 (−3.64, 2.57)	0.457
	2018–2021	9.98 (6.96, 12.80)	< 0.001
	2021–2023	−7.53 (−11.55, −3.52)	< 0.001
65–74	1999–2004	−1.51 (−3.32, 2.79)	0.185
	2004–2010	−4.75 (−8.24, 0.19)	0.053
	2010–2018	1.50 (−3.37, 2.42)	0.260
	2018–2021	5.18 (2.61, 6.94)	0.006
	2021–2023	−3.34 (−6.63, 0.40)	0.078
75–84	1999–2005	−1.85 (−2.85, 0.32)	0.069
	2005–2009	−6.24 (−8.71, −4.03)	0.010
	2009–2023	−0.35 (−0.72, 0.15)	0.147
≥85	1999–2012	−4.34 (−6.23, −3.70)	< 0.001
	2012–2023	−1.83 (−2.76, 1.02)	0.097
Race/ethnicity			
NH white	1999–2010	−3.39 (−4.05, −2.86)	< 0.001
	2010–2023	0.63 (0.21, 1.14)	0.005
NH black	1999–2005	−3.42 (−4.61, −0.44)	0.041
	2005–2012	−6.62 (−9.94, −5.46)	0.014
	2012–2023	−0.02 (−0.69, 0.74)	0.992
Hispanic/Latino	1999–2005	−1.86 (−4.99, 7.46)	0.394
	2005–2008	−9.82 (−12.97, 4.23)	0.172
	2008–2023	−0.23 (−4.29, 1.54)	0.695
NH Other (AI/AN, Asian/PI)	1999–2010	−5.55 (−13.20, −3.32)	0.002
	2010–2023	−0.54 (−1.88, 4.21)	0.751
Geographic region			
Northeast	1999–2010	−4.11 (−6.74, −3.05)	< 0.001
	2010–2023	−0.58 (−1.48, 1.29)	0.355
Midwest	1999–2010	−3.55 (−4.98, −2.71)	< 0.001
	2010–2023	0.44 (−0.22, 1.48)	0.166
South	1999–2012	−3.77 (−4.61, −3.17)	< 0.001
	2012–2023	1.28 (0.51, 2.32)	0.004
West	1999–2006	−1.95 (−3.07, 1.51)	0.086
	2006–2009	−5.68 (−7.13, 1.45)	0.096
	2009–2023	−0.27 (−1.36, 0.49)	0.282
**Urban–rural status**			
Large metropolitan	1999–2004	−2.88 (−4.22, 0.10)	0.053
	2004–2010	−5.47 (−8.21, −1.58)	0.029
	2010–2020	−0.47 (−1.10, 0.34)	0.175
Medium/small metropolitan	1999–2011	−2.99 (−5.91, −2.05)	0.004
	2011–2020	0.71 (−0.75, 5.65)	0.266
Rural	1999–2010	−2.95 (−4.56, −2.03)	< 0.001
	2010–2020	1.62 (0.57, 3.61)	0.004

APC, annual percent change; AAMR, age-adjusted mortality rate; CI, confidence interval; PAD, peripheral artery disease; NH, non-Hispanic; AI/AN, American Indian/Alaska Native; PI, Pacific Islander.

APCs (95% CIs) were estimated from log-linear joinpoint regression of annual rates (AAMRs for overall, sex, race/ethnicity, region, and urbanization strata; crude rates for age groups).

Urbanization-stratified joinpoint estimates were available through 2020 in CDC WONDER.

Across the study period, mortality was consistently higher among males than females (period-average AAMR, 3.96 vs. 2.76 per 100,000; Table 1). Long-term declines were observed in both sexes, with a larger AAPC decline among females (−1.99% per year) than males (−1.38% per year; Table 1; Figure 1B). Sex-specific joinpoint reference years were 2010 for males and 2012 for females (Figure 1B).

### Age gradient and age-specific temporal patterns

**Figure 2:**
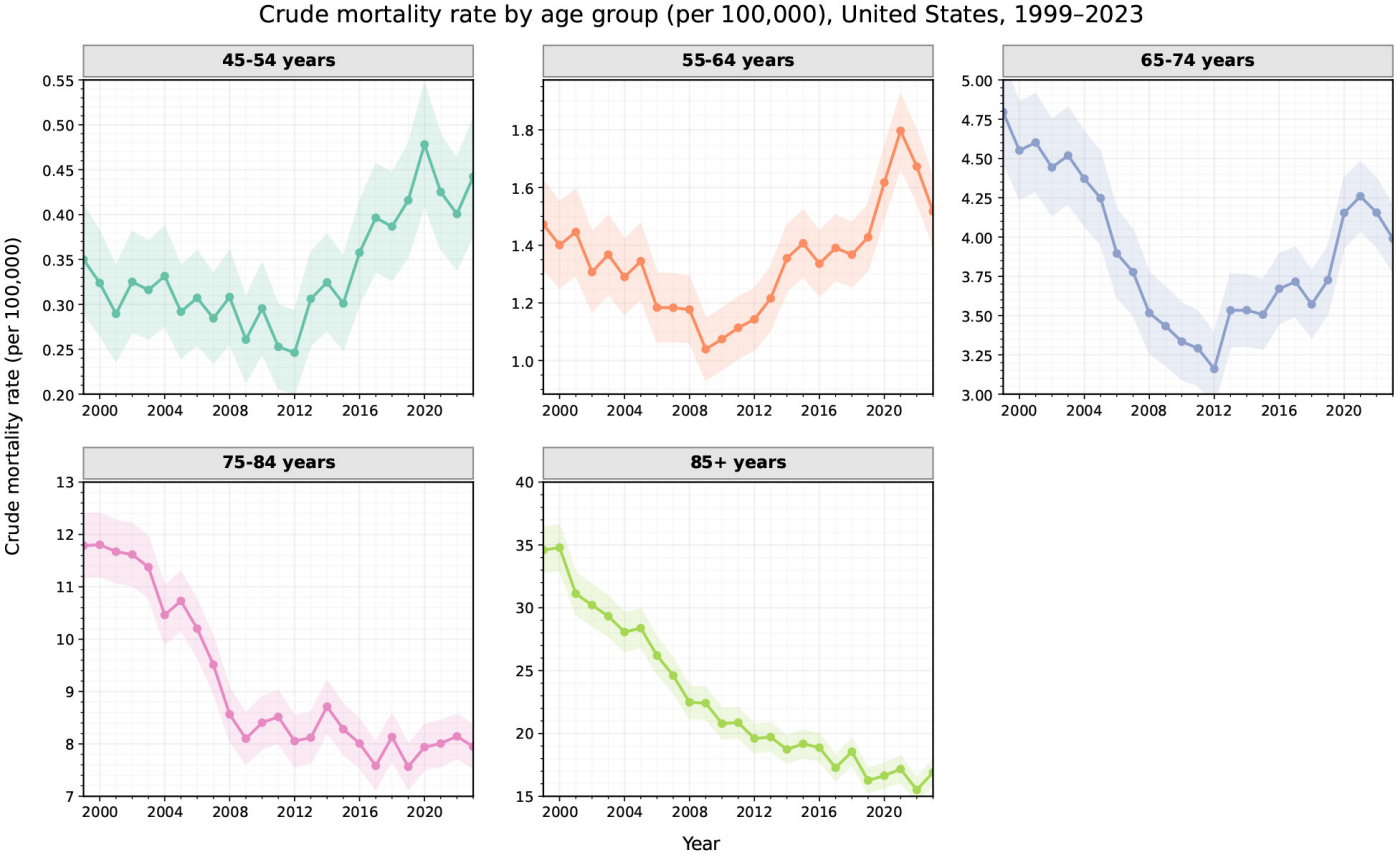
Crude mortality rate for PAD- and sepsis-associated mortality by age group, United States, 1999–2023. Annual crude mortality rates (per 100,000 population) are shown for five age strata (45–54, 55–64, 65–74, 75–84, and ≥85 years) with 95% confidence intervals. Points denote yearly estimates connected by lines. Each panel uses its own *y*-axis range to accommodate differences in rate magnitude across age groups.

Crude mortality rates increased steeply with age throughout the study period (Figure 2). Adults aged 85 years or older had the highest period-average crude rate (20.71 per 100,000), whereas adults aged 45–54 years had the lowest (0.33 per 100,000; Table 1). In older age strata, crude rates declined substantially over time (e.g., among adults aged ≥85 years, from 34.59 per 100,000 in 1999 to 16.90 per 100,000 in 2023; AAPC, −3.20% per year). In contrast, younger adults showed stable-to-increasing patterns (e.g., ages 45–54 years, from 0.35 per 100,000 in 1999 to 0.44 per 100,000 in 2023; AAPC, +0.91% per year; Table 1; Figure 2). The magnitude of decline increased progressively with age (AAPC range, −0.69% to −3.20% per year for ages 65–74 through ≥85 years).

### Rural–large metropolitan disparity

**Figure 3:**
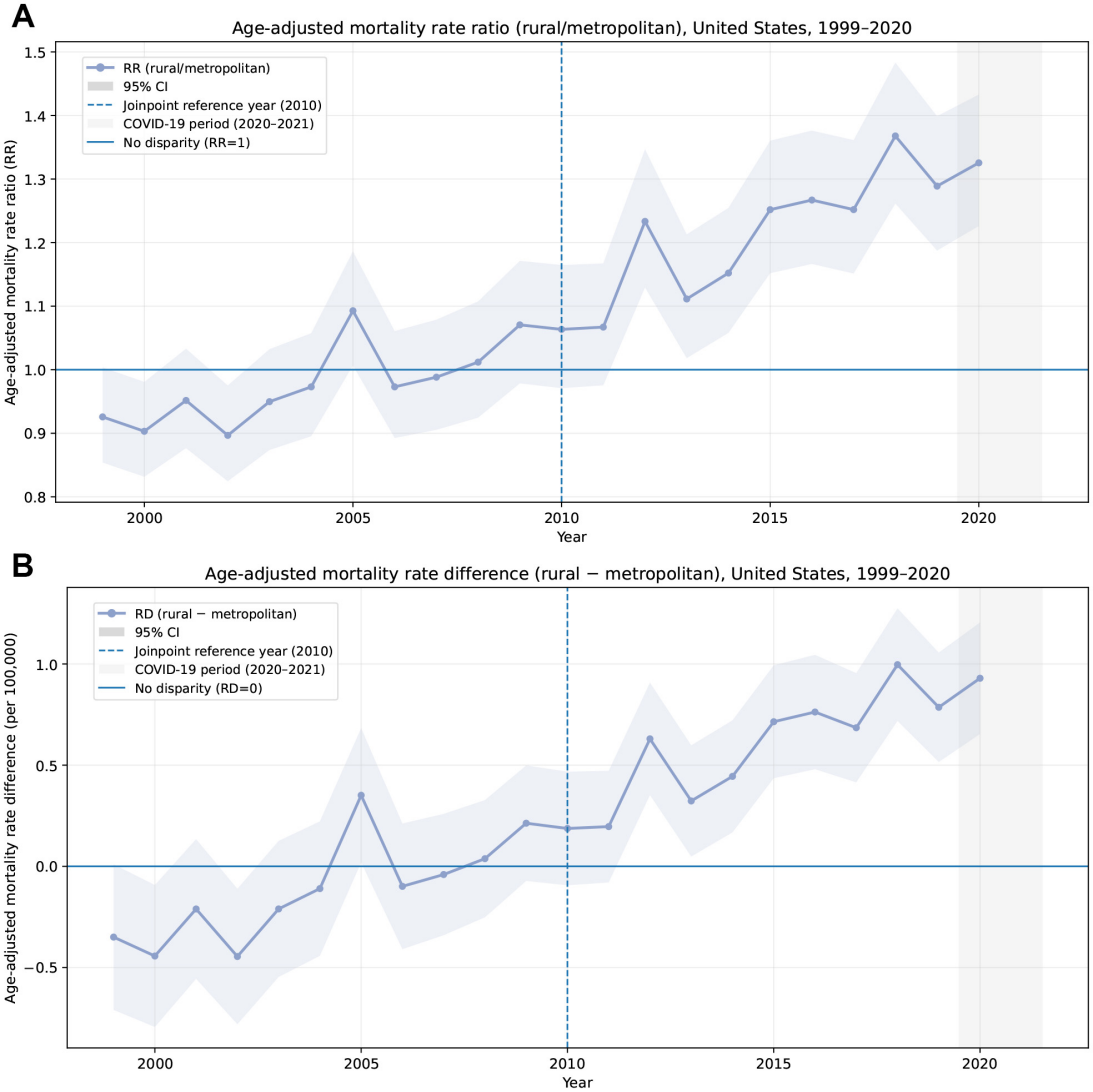
Rural–large metropolitan disparity in PAD- and sepsis–associated mortality, United States, 1999–2020. **(A)** The age-adjusted mortality rate ratio (RR, rural/large metropolitan) is shown with 95% confidence intervals. The horizontal reference line denotes no disparity (RR = 1). **(B)** The age-adjusted mortality rate difference (RD, rural–large metropolitan; per 100,000 population) is shown with 95% confidence intervals. The horizontal reference line denotes no disparity (RD = 0). In both panels, the dashed vertical line indicates the joinpoint reference year (2010), and the shaded band marks the COVID-19 period (2020–2021; only 2020 is shown).

Urbanization-stratified AAMRs were available for 1999–2020; therefore, rural–large metropolitan disparity analyses were restricted to this period. Rural–metropolitan disparities in AAMR changed over time (Figure 3). In 1999–2004, rural areas had similar or lower mortality than large metropolitan areas (RR < 1.0 with negative RD), with intermittent crossover in the mid-to-late 2000s. Beginning in 2010, disparities consistently favored large metropolitan areas, with higher rural mortality. Averaged over 2010–2019, the mean RR was 1.21 and the mean RD was 0.57 per 100,000 population. The disparity peaked in 2018 (RR, 1.37; RD, 1.00 per 100,000) and remained elevated in 2020 (RR, 1.33; RD, 0.93 per 100,000; Figure 3). Although rural areas had similar or lower AAMRs than large metropolitan areas during much of the pre-2010 period, the early rural deficit was modest and was outweighed by the sustained and larger rural excess after 2010. This later divergence explains why the period-average AAMR was higher in rural areas than in large metropolitan areas, despite the early-period crossover pattern. Annual RR and RD estimates with 95% CIs are provided in Supplementary Table S2.

### Race/ethnicity and regional patterns

**Figure 4:**
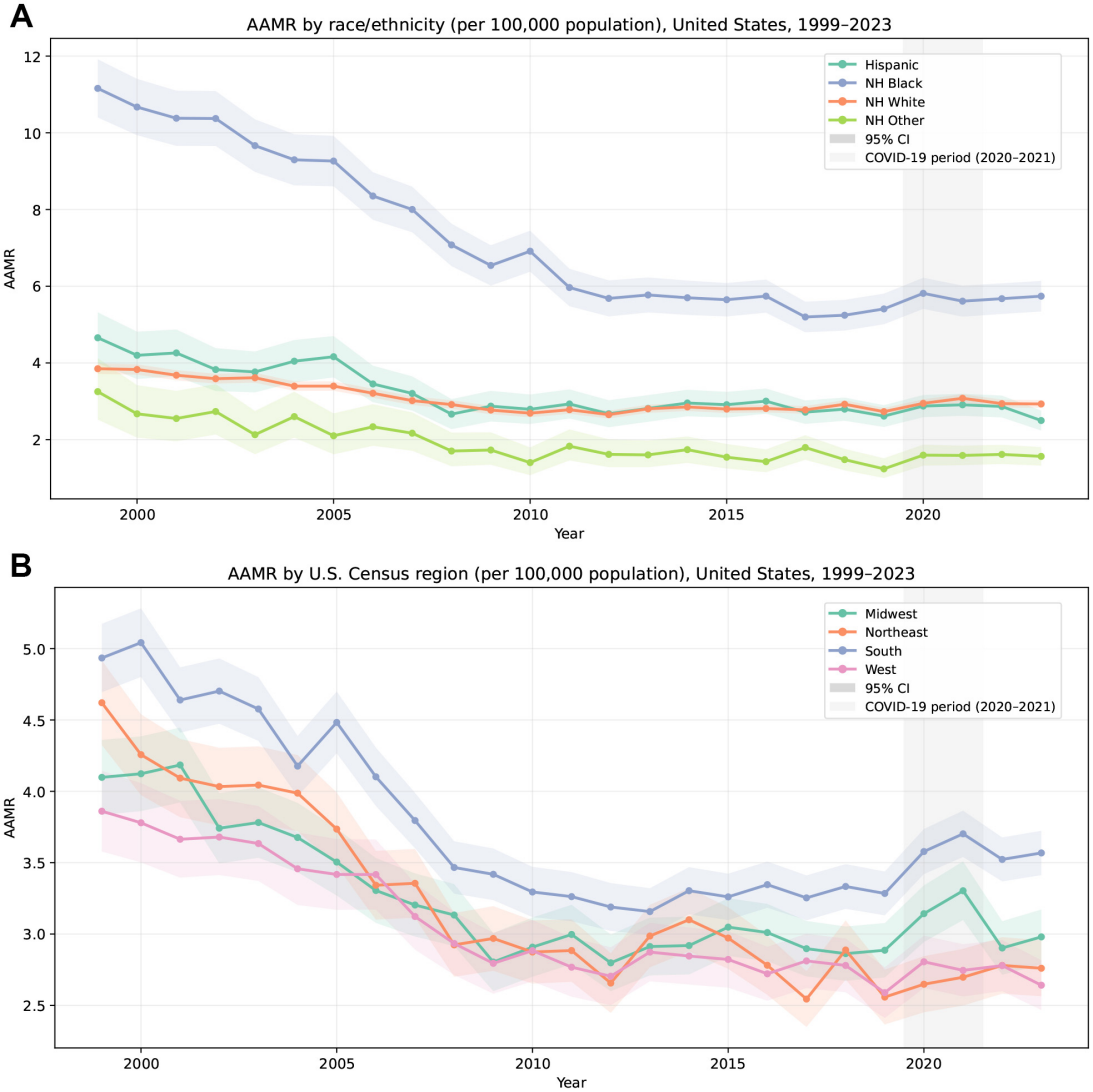
AAMR for PAD- and sepsis-associated mortality by race/ethnicity and U.S. Census region, United States, 1999–2023. **(A)** Annual age-adjusted mortality rates (AAMRs; per 100,000 population) are shown for Hispanic/Latino, non-Hispanic (NH) Black, NH White, and NH Other populations with 95% confidence intervals. **(B)** Annual AAMRs (per 100,000 population) are shown for the Midwest, Northeast, South, and West with 95% confidence intervals. Points denote yearly estimates connected by lines, and the shaded band marks the COVID-19 period (2020–2021).

Substantial racial and ethnic disparities were observed (Figure 4A; Table 1). NH Black individuals experienced the highest mortality burden (period-average AAMR, 6.48 per 100,000), exceeding that of NH White individuals (3.02 per 100,000) by more than twofold. Despite the higher burden, NH Black mortality declined markedly over time, from 11.16 per 100,000 in 1999 to 5.74 per 100,000 in 2023 (AAPC, −2.84% per year; Table 1; Figure 4A). NH Other had the lowest period-average burden (1.68 per 100,000; Table 1).

Regional variation was also evident (Figure 4B; Table 1). The South had the highest period-average AAMR (3.63 per 100,000), whereas the West had the lowest (2.96 per 100,000). All four Census regions demonstrated long-term declines, with the most rapid decrease in the Northeast (AAPC, −2.21% per year; Table 1).

### State-level heterogeneity

**Figure 5:**
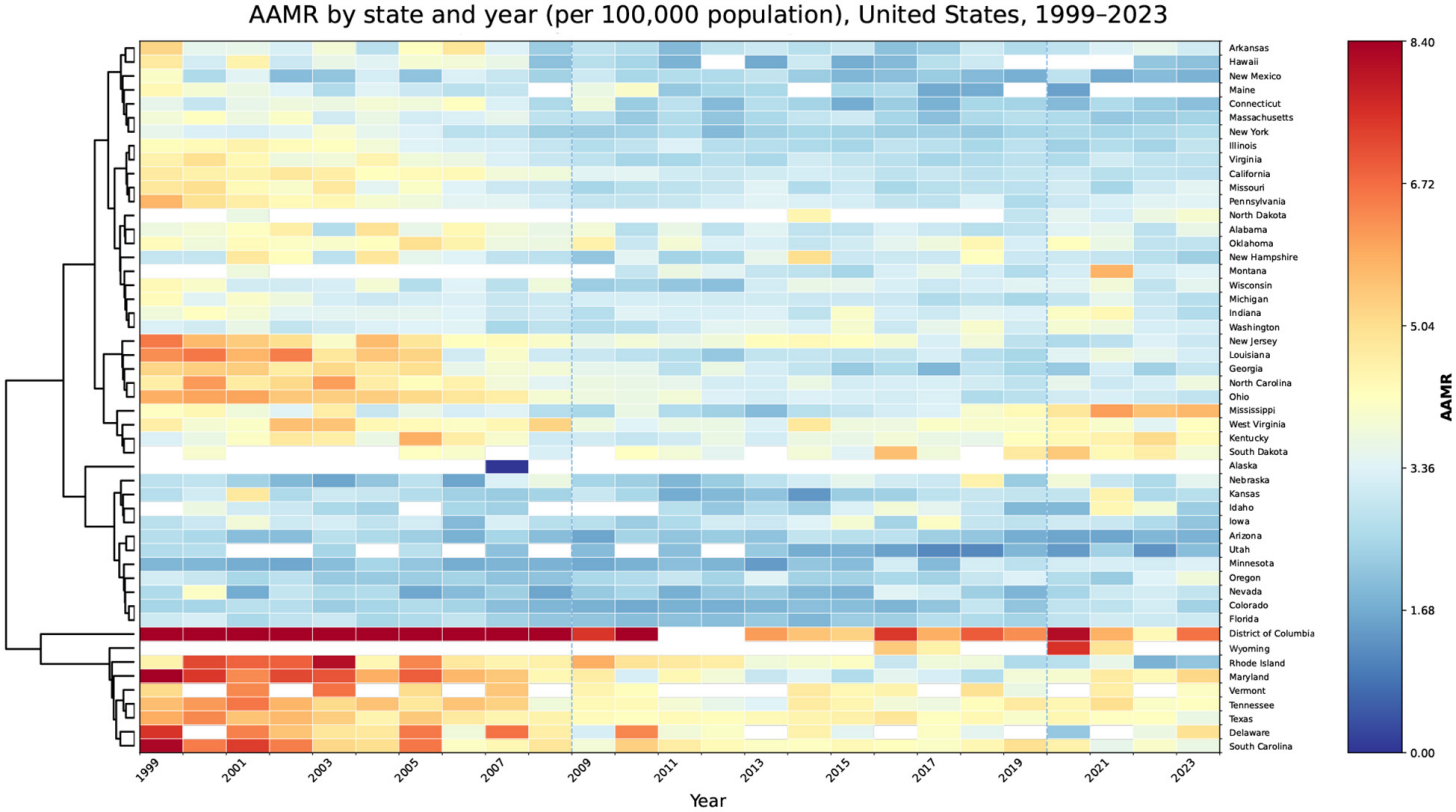
AAMR for PAD- and sepsis-associated mortality by state and year, United States, 1999–2023. Annual state-level age-adjusted mortality rates (AAMRs; per 100,000 population) are displayed as a heat map. States are ordered by hierarchical clustering (Ward linkage with Euclidean distance) based on similarities in temporal AAMR profiles; the dendrogram depicts the clustering structure. Cell color represents AAMR using a fixed scale (0.00–8.40); values above 8.40 are displayed at the upper color limit. White cells indicate missing estimates.

State-by-year heat maps revealed substantial geographic heterogeneity and clustering of states with similar temporal AAMR profiles (Figure 5; Supplementary Table S3). Several Southern states exhibited persistently higher AAMRs across multiple years (e.g., South Carolina, Tennessee, and Texas), whereas lower AAMRs were observed in states such as Arizona and Colorado (Figure 5). White cells indicate years with missing or suppressed estimates. Color intensity reflects AAMR magnitude using a fixed scale (0.00–8.40 per 100,000).

### Forecasts

**Figure 6:**
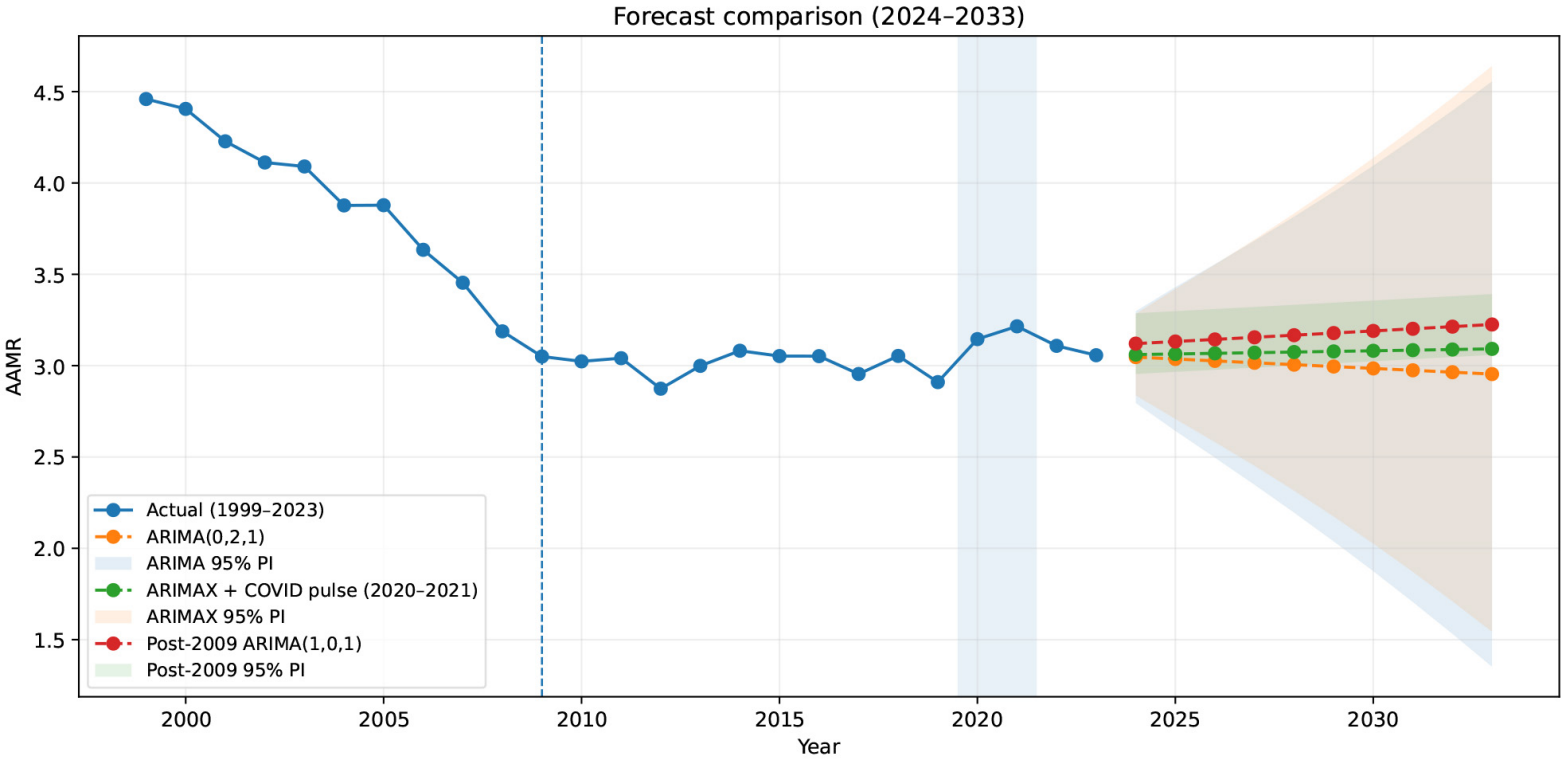
Forecast comparison of age-adjusted mortality rate (AAMR) for PAD- and sepsis-associated mortality, United States, 1999–2033. Annual AAMRs (per 100,000) for the overall population are shown for 1999–2023 (solid line with points), with forecasts for 2024–2033 generated using three approaches: (1) baseline ARIMA (0,2,1) model, (2) ARIMAX incorporating a COVID-19 pulse intervention term for 2020–2021, and (3) a joinpoint-aware segmented model fitted to the 2010–2023 period (shown as 2010–2023 joinpoint-aware ARIMA). Shaded bands represent 95% prediction intervals. The dashed vertical line indicates the joinpoint reference year (2009), and the shaded band marks the COVID-19 period (2020–2021).

Across forecasting specifications, projections for 2024–2033 suggested no rapid near-term escalation in PAD- and sepsis–associated mortality, although projected levels varied modestly by model (Figure 6). The baseline ARIMA (0,2,1) model projected a gradual decline in AAMR from 3.05 per 100,000 in 2024 to 2.95 per 100,000 in 2033. In contrast, the ARIMAX intervention model fitted to 1999–2023—with a prespecified COVID-19 pulse indicator (coded 1 for 2020–2021 and 0 otherwise, and set to 0 for 2024–2033 forecasts)—projected a slightly higher and nearly flat trajectory (3.06 in 2024 to 3.09 in 2033). The 2010–2023 joinpoint-aware approach (ARIMA [1,0,1] fitted to 2010–2023) yielded the highest projected levels and a slowly increasing trajectory (3.12 in 2024 to 3.23 in 2033; Figure 6).

Across all approaches, 95% prediction intervals widened with forecast horizon, reflecting increasing uncertainty over time (Figure 6). Model adequacy was supported by formal residual autocorrelation testing (Supplementary Table S4) and by visual inspection of residual ACFs for the baseline ARIMA and ARIMAX models (Supplementary Figure S1). Using the prespecified Ljung–Box test at lag 10, there was no evidence of residual autocorrelation for either full-series model (Supplementary Table S4). The estimated COVID-19 pulse coefficient in the ARIMAX model was positive but modest (Supplementary Table S4), consistent with a transient pandemic-era perturbation superimposed on the long-term trend rather than a sustained level shift.

## Discussion

In this population-based analysis of U.S. death certificate data from 1999 through 2023, we characterized national and subnational mortality patterns in which both PAD and sepsis were recorded as underlying or contributing causes of death. We identified temporal inflection points, quantified disparities across demographic and geographic strata, and generated near-term projections using complementary time-series approaches.

From a public health surveillance perspective, co-documentation of PAD and sepsis on death certificates provides a scalable indicator that integrates chronic vascular vulnerability with severe infection outcomes and can help track equity-relevant signals over time.

Overall, PAD- and sepsis-associated age-adjusted mortality declined over the 25-year period, with a transient increase during the prespecified COVID-19 interval followed by attenuation. Mortality was consistently higher among males than females, rose steeply with age, and demonstrated divergent age-specific trajectories—substantial declines among older adults but stable-to-increasing rates among individuals aged 45–54 years. Rural–metropolitan disparities widened after 2010, racial/ethnic inequities persisted (with the highest burden among NH Black individuals despite marked long-term improvement), and state-level patterns revealed pronounced geographic clustering. Across multiple forecasting specifications, projections suggested relative stability over the next decade, without evidence of rapid near-term escalation, although uncertainty increased with longer prediction horizons.

These findings are consistent with broader shifts in cardiovascular and infection-related outcomes that have occurred alongside changes in risk factor profiles, preventive care, acute-care capacity, and clinical recognition (1, 6, 23–25). The long-term decline in PAD- and sepsis-associated mortality is directionally concordant with secular improvements in cardiovascular risk management and broader uptake of evidence-based therapies for atherosclerotic disease, reductions in smoking, together with advances in early sepsis recognition and resuscitation (26, 27). The pandemic-era increase likely reflects converging mechanisms, including higher infection burden, disruptions in routine care for chronic vascular disease, delayed presentation, and strained hospital capacity (28–30). Our findings align with recent CDC WONDER-based analyses showing that overall sepsis mortality remained relatively stable from 1999 to 2019 (AAMR 77.51 to 76.1 per 100,000) but increased sharply by 30.22% from 2019 to 2021, primarily driven by COVID-19 (31). The modest COVID-19 pulse estimate and the attenuation after 2021 support the interpretation that this increase represented a perturbation superimposed on preexisting trends rather than a sustained level shift. Persistently higher mortality among males parallels established sex differences in PAD prevalence, cardiometabolic risk, and care-seeking patterns and may also reflect differential exposure to severe infections and treatment pathways (2, 6). Notably, the unfavorable trajectory among younger adults contrasts with declines in older age strata and may signal a shifting epidemiology in which earlier-onset cardiometabolic disease, diabetes, obesity, and other upstream determinants increasingly interact with severe infection risk (32), underscoring the need for targeted clinical and public health attention beyond the traditional emphasis on advanced-age PAD.

A central contribution of this work is its integrated examination of PAD and sepsis as co-occurring causes of death, rather than treating chronic atherosclerotic disease and severe infection as separate domains. Prior population studies have often examined PAD mortality, sepsis mortality, or related amputation and hospitalization outcomes independently (2, 3, 10). By requiring concurrent documentation of both conditions, our approach focuses on a clinically plausible intersection: individuals with systemic infection and concomitant atherosclerotic burden or impaired limb perfusion, in whom reduced physiologic reserve, microvascular dysfunction, and comorbidity clustering may amplify vulnerability (6, 7, 33). This perspective complements analyses based solely on hospital discharge diagnoses, which can be influenced by admission thresholds, coding priorities, and survival bias (3, 24). Methodologically, the combined use of joinpoint regression to detect inflection periods and time-series models to generate near-term projections offers a pragmatic surveillance framework: joinpoint methods help distinguish sustained secular changes from shorter-lived disruptions, whereas forecasts can inform anticipatory planning and hypothesis generation, particularly when interpreted alongside widening prediction intervals (19, 21).

The emergence and persistence of excess rural mortality after 2010 extends prior observations that rural populations experience disproportionate burdens of both cardiovascular disease and severe infections (15, 34). Several mechanisms may contribute, including delayed access to vascular specialists, limited availability of endovascular and surgical limb-salvage services, fewer intensive care resources, and longer transport times—factors that may compound the risk of severe infection and adverse outcomes among individuals with PAD (35, 36). Structural conditions, such as higher prevalence of diabetes and smoking, lower insurance coverage in some regions, healthcare workforce shortages, and hospital closures, may further widen this gap (23, 36). The peak rural–metropolitan differential in the late 2010s and its persistence into 2020 suggest that inequities were entrenched before the pandemic and may have been magnified by pandemic-related strain, emphasizing that stabilization of aggregate rates can still conceal substantial and durable disparities (29, 37).

Racial and ethnic differences were also pronounced. The persistently higher burden among NH Black individuals, despite substantial improvement over time, aligns with the well-documented effects of structural inequities, differential exposure to cardiometabolic risk factors, barriers to preventive and specialty care, and unequal access to timely, high-quality acute care (10, 25, 38). The steeper decline in mortality among NH Black individuals relative to NH White individuals suggests meaningful gains, yet the remaining excess highlights persistent gaps. State-level heterogeneity—particularly higher burdens in several Southern states—likely reflects geographic clustering of cardiometabolic risk, place-based barriers to healthcare access, and variation in health system capacity (2, 38). Together, these patterns indicate that the PAD–sepsis interface is shaped not only by individual-level biology and comorbidity but also by place-based determinants and health system factors.

This study has several limitations. First, it relies on death certificate data, which are subject to misclassification of both PAD and sepsis and to temporal changes in diagnostic recognition and coding practices. Because we defined cases using ICD-10 codes listed anywhere on the certificate, ascertainment may vary across jurisdictions and over time, potentially influencing trend estimates. In addition, our PAD definition prioritized phenotype specificity by including I70.2 and I73.9 while excluding broader atherosclerosis codes such as I70.0, I70.1, I70.8, and I70.9. This conservative approach may underestimate PAD- and sepsis-associated mortality when peripheral disease was coded only under generalized or less specific atherosclerosis codes; however, including these broader codes could have introduced non-peripheral atherosclerotic disease and weakened the specificity of the PAD–infection phenotype examined in this study. Similarly, the PAD definition did not include I73.1 for thromboangiitis obliterans/Buerger's disease. Although Buerger's disease may share clinically relevant features with PAD, including limb ischemia, skin breakdown, and infection susceptibility, it represents a distinct non-atherosclerotic inflammatory vasculopathy; therefore, its exclusion may underestimate the broader burden of peripheral ischemic conditions co-documented with sepsis, while its inclusion would have reduced the etiologic homogeneity of the primary PAD phenotype. Second, the multiple-cause-of-death database lacks granular clinical detail (e.g., PAD severity, infection source, microbiology, treatment timing, hospital course, revascularization history, and comorbidity burden), limiting mechanistic inference and precluding causal attribution regarding pathways linking PAD to sepsis-related death. Third, CDC WONDER suppression of small cell counts may disproportionately affect estimates for smaller states, rural strata, and some racial/ethnic groups, and race/ethnicity classification on death certificates can be imperfect—factors that may bias comparisons and understate disparities in certain populations (39). We partially mitigated these constraints by applying age adjustment, using standardized methods consistent with CDC WONDER, and deploying complementary modeling strategies (including an explicit COVID-19 pulse specification and post-joinpoint segmentation) to test the robustness of temporal interpretations. Nonetheless, residual bias from measurement error and suppression cannot be excluded. Future studies linking mortality data to clinical registries, claims, or hospitalization datasets could improve phenotyping and reduce misclassification. Fourth, urbanization-stratified AAMRs were available only through 2020, and rural–urban comparisons for 2021–2023 could not be evaluated. This limitation is particularly relevant because the prespecified COVID-19 period extended through 2021 and because rural populations may have experienced disproportionate pandemic-era strain related to delayed access, hospital capacity constraints, and workforce shortages. Consequently, the elevated rural excess observed in 2020 may underestimate the full pandemic-era rural burden, and future analyses using updated urbanization-linked mortality files are needed to determine whether rural disparities widened further, persisted, or attenuated after 2020.

Despite these limitations, the findings underscore a clinically and policy-relevant intersection between chronic atherosclerotic disease and severe infection. Prevention and management strategies may be more effective if they explicitly integrate vascular and infectious risk. For patients with PAD—particularly those with diabetes, chronic kidney disease, prior limb complications, or limited access to specialty care—more intensive risk-factor control, improved vaccination uptake, routine foot and skin surveillance, rapid evaluation of suspected infection, and clear escalation pathways may reduce progression to severe sepsis (1, 6, 40). As emphasized in the 2024 ESC guidelines for peripheral arterial and aortic diseases, comprehensive cardiovascular risk assessment and optimal medical therapy remain foundational to improving outcomes in this population (41). For population health impact, prevention strategies should be aligned with equity-focused delivery models, particularly in rural and high-burden states.

Health systems could strengthen integrated care models that link outpatient PAD management with standardized sepsis screening and early treatment protocols in emergency and inpatient settings (42). Efforts to reduce delays in rural settings may include telemedicine-enabled triage, regional transfer pathways, and support for critical access hospitals (35, 43). From a surveillance perspective, combining death certificate–based indicators with hospitalization data may help distinguish true incidence changes from documentation effects and identify modifiable clinical touchpoints (e.g., time to antibiotics, access to revascularization, and post-discharge follow-up). Finally, reducing disparities will likely require place-based investments that expand access to preventive care, strengthen workforce capacity, and address social determinants that cluster in high-burden states and rural communities (25, 36).

Several lines of inquiry follow from these results. First, linkage studies should evaluate whether observed trends primarily reflect changes in PAD prevalence and severity, sepsis incidence, case fatality, or documentation practices, and should assess how these components vary by age, race/ethnicity, and geography. Second, analytic extensions incorporating county-level socioeconomic indicators, healthcare access metrics (e.g., ICU bed availability, vascular specialist density, exposure to hospital closures), and comorbidity profiles could clarify drivers of the rural and Southern excess and identify actionable targets. Third, mechanistic research is needed to define the clinical phenotype of patients with PAD who develop severe infection, including the relative contribution of limb- vs. non-limb-related sources, pathogen patterns, and the role of chronic inflammation, microvascular dysfunction, and impaired tissue perfusion (44). Fourth, the flat or unfavorable trends among younger adults warrant focused investigation of upstream determinants (including cardiometabolic risk trajectories and healthcare engagement) and evaluation of prevention strategies tailored to earlier-life risk. Finally, ongoing surveillance should assess whether post-pandemic patterns stabilize, improve, or worsen and whether interventions targeting equity and access translate into measurable reductions in PAD- and sepsis-associated mortality at the population level.

## Conclusions

In this nationwide analysis of U.S. multiple-cause-of-death data from 1999 through 2023, mortality with co-documented PAD and sepsis declined overall, with a transient increase during the COVID-19 period followed by attenuation. Nonetheless, substantial inequities persisted, including higher mortality among males, an unfavorable trajectory in adults aged 45–54 years, an emergent and sustained rural excess after 2010, the highest burden among NH Black individuals despite marked improvement, and pronounced state-level clustering. These findings delineate a clinically meaningful PAD–sepsis intersection and support integrated prevention and care strategies that bridge vascular risk management with infection prevention, early detection, and timely treatment, particularly in disproportionately affected communities and regions. Ongoing surveillance linking mortality patterns with clinical and hospitalization data will be essential to clarify mechanisms, distinguish epidemiologic change from documentation effects, and evaluate equity-focused interventions aimed at reducing population-level mortality.

## Data Availability

The original contributions presented in the study are included in the article/Supplementary material, further inquiries can be directed to the corresponding authors.

## Supplementary material

Supplementary Figure S1 Residual autocorrelation function (ACF) for baseline ARIMA and ARIMAX models, United States, 1999–2023. Residual ACFs are shown for post-fit model residuals from the baseline ARIMA (0,2,1) model and an ARIMAX model incorporating a prespecified COVID-19 pulse intervention (indicator coded 1 for 2020–2021 and 0 otherwise) fitted to the overall annual AAMR series (1999–2023).

Supplementary Table S1 ICD-10 code definitions and analytic strata (CDC WONDER MCOD, United States, 1999–2023).

Supplementary Table S2 Annual rural vs. large metropolitan disparities in AAMR, United States, 1999–2020.

Supplementary Table S3 State-level burden and temporal trends in PAD- and sepsis–associated mortality (United States, 1999–2023).

Supplementary Table S4 Forecast model comparison for overall AAMR (United States, 1999–2023; forecasts 2024–2033).
