# Supplementary figures and images for: Trends, disparities, and forecasts of peripheral artery disease- and sepsis-associated mortality in the United States, 1999–2023
Source: Front Med (Lausanne). 2026 Jul 2;13:1844478. doi: 10.3389/fmed.2026.1844478 (PMC13373651; doi:10.3389/fmed.2026.1844478)

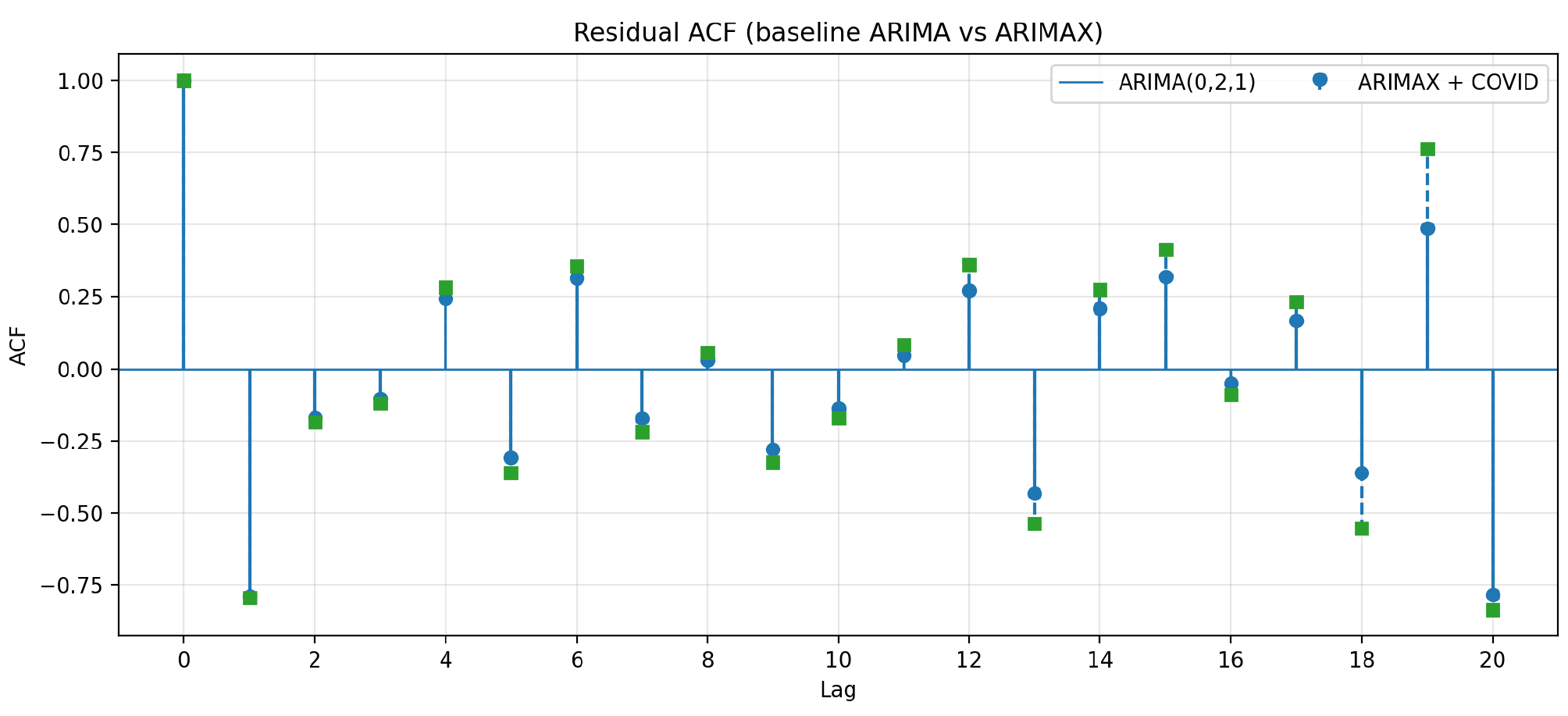

Supplement: Supplementary Figure S1 — Residual autocorrelation function (ACF) for baseline ARIMA and ARIMAX models, United States, 1999–2023. Residual ACFs are shown for post-fit model residuals from the baseline ARIMA (0,2,1) model and an ARIMAX model incorporating a prespecified COVID-19 pulse intervention (indicator coded 1 for 2020–2021 and 0 otherwise) fitted to the overall annual AAMR series (1999–2023). [file Image_1.tif]
